# The Territorial Medical Schools of Instruction

**Published:** 1908-09-26

**Authors:** 


### The Royal Army Medical Corps Section.

### THE TERRITORIAL MEDICAL SCHOOLS OF INSTRUCTION.

Recognising the fact that the efficiency of the Medical Service of the Territorial Army cannot be attained simply by organisation, but that there is required in addition a proper system of training for officers, non-commissioned officers, and men, the military authorities must have credit for another praiseworthy attempt to solve this very important problem. A new and very commendable departure they have made in the matter is to establish Territorial Medical Schools of Instruction. Although on a smaller scale, they correspond to the R.A.M.C. Training Depôt and School of the Regular Army at Aldershot, where there exists a complete staff of instructors in all the branches of work, through whose hands all the raw material passes until, examined and inspected, it is drafted to the companies of the R.A.M.C. By such a plan the men of the corps pass through one and the same channel, and all are instructed on a uniform basis.

Before giving an account of the Territorial Schools of Instruction, it may be as well to draw attention to one or two excellent principles embodied in the recently issued scheme of training for the R.A.M.C. (T). One is that while common instruction may very well mark the early stage of training, as when the very essential knowledge of drill and first-aid is being inculcated, as soon as the tuition in these necessary but elementary subjects is completed, the individuals should be classified according to the duties they are meant to carry out, and a differentiation of work instituted. In this way the *personnel* of the nursing, cooking, clerical, and general duty sections are from an early date trained in their own special lines of work. Another commendable feature in the scheme of training is that it is framed to ease personal liability as much as possible. By the plan of alternative courses there is brought into play that considerate elasticity which is so necessary in connection with the demands of civilian life. Such courses may take the place of the required yearly attendances for drill and instruction at the headquarters of a man's unit, or they may replace his attendance at camp, this latter duty being in no case obligatory on any member of the R.A.M.C.(T.). Further, these alternative courses may be attended at more than one place, the choice lying between a selected military hospital, a recognised civilian hospital, the R.A.M.C. Training School at Aldershot, and an R.A.M.C.(T.) School of Instruction.

The authority for the formation of the Territorial Medical Schools of Instruction is shown in the special Army Order dated March 18, 1908, Table D. They will be located at the headquarters of selected field ambulances, and the senior commanding officer of the field ambulance will be the commandant of the school. The permanent staff of the school will consist of an adjutant and three instructors, all belonging to the R.A.M.C. Of the three instructors, two will be for ordinary corps instructional purposes, one of whom will act as sergeant-major, and the third one will be for technical medical transport duties. The work of the adjutant and the permanent staff will consist in training the recruits of the R.A.M.C.(T.) in drill and first-aid before they are finally passed on to their respective units, but the commanding officers of these units, although receiving this general assistance and help in the training of their men, are in no way relieved of the duty of training them. They are held entirely responsible for the efficiency of their units. In addition to the above, it will fall to the staff of the school to train officers in the various duties connected with the administrative work of medical units, and to conduct special courses in sanitation, hygiene, first-aid, nursing, dispensing, and cooking for non-commissioned officers and men who require to pass in these subjects for promotion, or to take them as alternatives to annual attendance or camp training. These courses at the schools of instruction are of eight days' duration, and they embrace theoretical instruction along with such practical work as can be arranged.

While, however, the staff of a Territorial School of Instruction consists permanently of an adjutant and three instructors, there has very properly been given to the commandant of the school the power to add to the instructional staff if he considers it necessary. He can temporarily augment it so as to provide interesting and useful subjects for study other than those laid down. In fact, a commandant vested with such powers, if he is a man of enterprise and initiative, can make his school a powerful influence for good in several ways. Regarding the authorised staff as a nucleus round which there can be grouped a valuable body of outside workers, he might enlist the service of members of the medical and nursing professions, who would be willing, although unable, to enrol in the Territorial Force, to deliver courses of lectures at stated intervals on special subjects connected with the instruction of the different nursing and other sections of the R.A.M.C.(T.). Especially in the matter of sanitation would this outside help be of great service, and we feel sure that from medical officers of health and other experts in sanitary science instructional assistance would be readily forthcoming. We are also glad to observe that these schools of instruction for the Territorial Force are to have a certain amount of mobility. They are not to be rigidly anchored down at the headquarters where they are located, but may be temporarily employed for instruction at the headquarters of other units in the division to which they belong; or, in the case of areas which provide two divisions for the Territorial Force, at the headquarters of units in either division.

By all members of the Territorial Medical Service the establishment of these schools of instruction should be warmly welcomed. They will, to a large extent, do away with the difficulties which hitherto existed in connection with the old Volunteer Medical Service and prevented medical officers obtaining certain certificates of proficiency, for they furnish to officers, non-commissioned officers, and men those facilities for training in the various branches of medical work, without which qualifying examinations cannot be successfully passed. We have already on a previous occasion expressed our satisfaction at the reasonable and considerate tone pervading the conditions of qualifying service and of training laid down for the members of the Territorial Medical Service, and this attitude on the part of the military authorities should bear good fruit. The Medical Service is a branch of our Territorial Army that can be of the greatest service and that has much to render it attractive; and if those enrolling feel that they are given facilities in their work, they will quickly recognise it and induce others to join. Another fact that should not be lost sight of by those in authority is that these schools will fail in their object if they are stinted of instructors and not fully equipped with suitable buildings and the latest pattern of equipment. Territorialists may not be professional soldiers, and may be regarded as amateurs for whom the old-fashioned and cast-off equipment of the Regular Army may be considered good enough, but they are not wanting in intelligence, and they will not waste their time in drilling with discarded articles that will not allow them to obtain that proficiency they are anxious and willing to acquire and that can alone render them useful in the field. A great deal of the so-called inefficiency of the old Volunteer Force was due to the issue of obsolete equipment, and it is to be hoped the country will insist on this unfair line of treatment being at once abandoned.

